# Introducing the *Journal of Cognition*

**DOI:** 10.5334/joc.1

**Published:** 2017-08-09

**Authors:** Robert J. Hartsuiker, Candice C. Morey

**Affiliations:** 1 Department of Experimental Psychology, Ghent University, BE; 2 Department of Psychology, University of Edinburgh, GB

We are thrilled to present the *Journal of Cognition (JoC)*, the new official journal of the European Society for Cognitive Psychology (ESCoP). The submission portal has been online since 19 May 2017 and the editorial team has already started to handle the first submissions. With the startup of JoC, ESCoP says farewell to the *Journal of Cognitive Psychology (JCP)*, which – initially under the title *European Journal of Cognitive Psychology (EJCP)* – has been ESCoP’s official journal since 1989. In this opening editorial, we briefly look back at the history of EJCP/JCP, explain the decision to discontinue with JCP, and present the role we hope JoC will play in the field of cognitive psychology.

## Looking back: The “European journal”

The EJCP started in 1989, four years after the foundation of ESCoP. EJCP has been indexed in ISI since 1992, and has had an impact factor that fluctuated between 0.42 (in 2000) and 1.89 (in 2015). One of the papers from the early days (Prinz, 1997) became a classic of the field with close to 1000 citations. In his 1989 opening editorial, Michael Eysenck expressed the hope that the journal would attract papers “from all countries of Europe”, but that submissions from “non-European countries were very welcome too”. To make this latter point more transparent, Janet Van Hell (Editor-in-Chief in the period 2009–2013) decided to drop “European” from the title. Since the title change in 2011, the journal has indeed become global: in 2016, 44% of submissions came from outside of Europe, especially North America and Asia.

## Changing views of research practices in psychology

During our term as Editor and Associate Editor of JCP (2013–2017) many psychological scientists began to reconsider the field’s research standards as psychology was confronted with several issues appearing to threaten the credibility of our discipline. These include low replication success (e.g., Open Science Collaboration, 2015), underpowered studies (e.g., Bakker, Van Dijk, & Wichters, 2012), publication bias (e.g., Francis, 2012), unwillingness or inability to share data (e.g., Vanpaemel, Vermorgen, Deriemaecker, & Storms, 2015), and use of questionable research practices (e.g., Simmons, Nelson, & Simonsohn, 2011). There was also debate about the ways we make statistical inferences, with increasing skepticism of traditional null-hypothesis significance testing (Wagenmakers, 2007). Many questions were raised as well about the publication practices in academia as dissatisfaction grew with the traditional business model, which pits researchers’ priorities of broad availability and openness against publishers’ motive to profit financially.

We felt that part of the responsibility for promoting good standards in our field lies with journal editors such as ourselves. We therefore decided upon two sets of actions. First, JCP adopted a set of methodological guidelines that were first introduced by the *Psychonomics* journals (http://www.psychonomic.org/?page=journals), and which explicitly draw attention to issues such as statistical power, pitfalls of multiple null hypothesis significance tests (including the issue of “running extra participants” if the test does not produce a p-value below .05), cherry-picking from multiple conditions, variables, experiments, or tests, and “harking” (hypothesis testing after the results were known). The guidelines further encourage authors to provide rich descriptions of their data and to use the statistical methods that “best describe and convey the properties of their data” (which does not necessarily mean classical null hypothesis significance testing).

Second, we introduced an Open Science policy for JCP. The journal became a signatory of the TOP guidelines https://cos.io/our-services/top-guidelines/, an initiative devoted to promoting transparency and openness in science. One essential element of openness in science is that authors, *by default*, make their data available to the reviewers and editor during the review process, and to the public after the paper is accepted. That is, in principle, data should be open, unless of course there are good reasons not to disclose them (and in this case these reasons should be given, as also described by the Peer Reviewers’ Openness Initiative; Morey, et al., 2016). This requirement has been in place at JCP since January 2017.

## Open access: one bridge too far?

JCP’s publisher, Taylor and Francis, were helpful and supportive with respect to the implementation of the aforementioned changes. However, Taylor and Francis and ESCoP did not reach an agreement with respect to our wish to break with the traditional publishing model and move towards open access. In 2016, the publisher proposed to add an annual open access supplement to the traditional journal, but imposed the condition that ESCoP should still fill an entire “traditional” volume each year. We worried that these two publishing models could not be simultaneously promoted, at least not forever. If we encouraged authors to publish in the open access supplement, we could have ended up with too few contributions for the traditional volume to satisfy our obligations to the publisher. After considerable discussion and negotiation, the ESCoP executive committee decided unanimously to not renew the contract with Taylor and Francis. As Taylor and Francis own the title “*Journal of Cognitive Psychology*”, they are free to continue the journal, but no longer under the auspices of ESCoP (to ensure ESCoP’s contractual obligations are fulfilled, RH and his team of associates stayed on until 1 April 2017, and will continue to deal with papers submitted before that date until they have a final disposition).

## Looking to the future: Journal of Cognition

We are very glad that with the start of JoC, ESCoP has regained control of its own official journal. The society, not the publisher, owns the title and the journal is completely open access. JoC will offer cognitive psychologists worldwide an open access outlet for their research, in a journal that strongly promotes excellent research practices (including open science), with strong quality control, and at a reasonable price for authors that reflects the real costs of evaluating and producing an article.

JoC’s board of editors is firmly committed to promoting excellent scientific standards and views an open science policy as intrinsic to the best research practices. Thus, JoC has signed the TOP initiative, has an open data policy, and has a set of guidelines promoting high standards in the analysis and reporting of data. We encourage the submissions of registered reports and data reports in addition to standard empirical and review articles.

JoC is committed to quality control. This is all the more important in a time when the open access model itself is under threat from questionable publication practices, which range from for-profit models with little incentive to weed out weak papers, to clearly predatory practices (e.g., Sorokowski, Kulczycki, Sorokowska, & Pisanski, 2017). We feel that the best guarantee for a system of Open Access, with strong quality control and with a democratic price for authors, is a model in which a journal is connected to a learned society rather than a commercial publisher. This is precisely JoC’s model, and so authors can expect all the benefits of Open Access publishing, along with the same thorough review process they are used to from JCP.

The transition from JCP to JoC is supported by all former associate editors of JCP; in fact, with the exceptions of Rob Hartsuiker (who is taking a break from editing after having edited for Acta *Psychologica*, *Psychological Science*, and *JCP* since 2004) and Matthias Kliegel (who begins a term as Editor-in-Chief for the *European Journal of Ageing* in the summer of 2017), the complete team of associate editors (Jos Adam, Linden Ball, Jelena Havelka, Mei-Ching Lien, Manuel Perea, and Orly Rubinsten) joined the founding editorial board of JoC. Additionally, several new associate editors have joined us, namely Pablo Gomez, Katharina Schnitzpahn, Bob Slevc, Hedderik van Rijn, and Bettina von Heleversen. We hope that the journal will attract submissions from every corner of the planet (as did JCP) and every sub-discipline in cognition, but that it will become an outlet of choice particularly for members of ESCoP.

Robert J. Hartsuiker, *outgoing editor, Journal of Cognitive Psychology.*

Candice C. Morey, *incoming editor, Journal of Cognition.*
